# Human factors in resuscitation: Lessons learned from simulator studies

**DOI:** 10.4103/0974-2700.70764

**Published:** 2010

**Authors:** S Hunziker, F Tschan, N K Semmer, M D Howell, S Marsch

**Affiliations:** 1 Medical Intensive Care Unit, University Hospital Basel, Basel, Switzerland; 2 Silverman Institute for Health Care Quality and Safety and the Department of Medicine, Beth Israel Deaconess Medical Center, Boston; 3 Harvard Medical School, Boston, MA, USA; 4 Department of Psychology, University of Neuchâtel, Switzerland; 5 Department of Psychology, University of Bern, Bern, Switzerland

**Keywords:** Cardiopulmonary resuscitation, leadership, team behavior

## Abstract

Medical algorithms, technical skills, and repeated training are the classical cornerstones for successful cardiopulmonary resuscitation (CPR). Increasing evidence suggests that human factors, including team interaction, communication, and leadership, also influence the performance of CPR. Guidelines, however, do not yet include these human factors, partly because of the difficulties of their measurement in real-life cardiac arrest. Recently, clinical studies of cardiac arrest scenarios with high-fidelity video-assisted simulations have provided opportunities to better delineate the influence of human factors on resuscitation team performance. This review focuses on evidence from simulator studies that focus on human factors and their influence on the performance of resuscitation teams. Similar to studies in real patients, simulated cardiac arrest scenarios revealed many unnecessary interruptions of CPR as well as significant delays in defibrillation. These studies also showed that human factors play a major role in these shortcomings and that the medical performance depends on the quality of leadership and team-structuring. Moreover, simulated video-taped medical emergencies revealed that a substantial part of information transfer during communication is erroneous. Understanding the impact of human factors on the performance of a complex medical intervention like resuscitation requires detailed, second-by-second, analysis of factors involving the patient, resuscitative equipment such as the defibrillator, and all team members. Thus, high-fidelity simulator studies provide an important research method in this challenging field.

## INTRODUCTION

It is well known that immediate and effective cardiopulmonary resuscitation (CPR) is of outmost importance to improve mortality and morbidity rates of patients after cardiopulmonary arrest.[1] Every minute that CPR is delayed decreases survival by up to 10%.[2] To ensure optimal performance of CPR, the International Liaison Committee on Resuscitation (ILCOR) has recognized several crucial elements[3] including clear resuscitation guidelines, optimal equipment and resources, and regular retraining of healthcare workers. Resuscitation training should not only include teaching of theoretical knowledge or memorized algorithms, but also more importantly should improve the hands-on skills of rescuers. As training in actual resuscitations exposes patients to risks and may traumatize inexperienced rescuers, simulator training is particularly appropriate for resuscitation.[4,5] It allows hands-on training in critical situations without these risks. Most importantly, a video-assisted debriefing allows learners to critically reflect on their performance in detail and can thus help to improve their skills. In addition, simulator sessions are a potentially important research tool to assess human behavior.

Although current resuscitation training guidelines[5] do not explicitly include the training of team-related aspects such as leadership or coordination, recent studies have stressed the potential importance of human factors for resuscitation performance and training,[3] and more generally, for reducing errors and improving safety in medicine.[6] However, there is still substantial debate on this issue, particularly in resuscitation.[3]

The aim of this paper is to summarize evidence from simulator studies assessing human factors and their influence on the performance of resuscitation teams. We will particularly focus on the research of an interdisciplinary simulator research team in Switzerland and discuss the following major points:

1. Simulator studies are an important new research method that allows rigorous assessment of complex interactions during serious and infrequent or Infrequently occurring emergencies in a realistic environment and without putting patients at risk.
2. Simulator studies of cardiac arrests demonstrate important delays in the initiation of guidelines-based life-saving measures (i.e., early defibrillation and initiation of CPR) and unnecessary interruptions of CPR. These findings mirror observations in real patients.
3. Because, in studies of real patients, logistical issues usually preclude observations of the first few moments of CPR, simulator studies offer great opportunities to investigate early team building and communication.
4. Simulator studies repeatedly show that human factors play a major role in both occurrence and avoidance of shortcomings in CPR performance. Adequacy of resuscitation was strongly influenced by the quality of leadership and team-structuring.
5. Simulator studies of medical emergencies reveal that a substantial part of the information transfer during communication is erroneous. Since the identification of erroneous information requires objective knowledge of patients’ characteristics and proceedings, analysis of video recordings of medical simulation allows the diagnosis of information failures. By contrast, the identification of erroneous information in real patients is very difficult and may be impossible.
6. There is evidence that medical simulation is suitable to investigate various aspects of human errors.

## SIMULATION AS AN OPPORTUNITY FOR STUDYING TEAM PERFORMANCE AND HUMAN FACTORS

In different high-reliability domains, such as aviation and high technology medical fields, simulator training has become a cornerstone of education and research.[7,8] Similarly, in high-risk medical situations, simulator training and research based on simulator sessions have become increasingly important for several reasons.[9] First, high-fidelity simulations allow training and assessment of complex skills in a realistic environment without putting patients at risk. Indeed, participants in simulator sessions rate them as highly realistic.[10,11] Second, simulation training allows presentation of low-frequency, high-severity situations: events that practitioners encounter only infrequently,[12] but where rapid and professional responses are crucial. Furthermore, video-assisted debriefing allows providers to review performance without time-pressure or relying on recollection of events, and thus seems to be very well suited for training purposes.[13] Besides training, simulators also offer good possibilities for research, because one can create identical study situations as well as experimentally controlled variations. Although most research done in simulators still concentrates on the evaluation of technical skills,[14] simulators are particularly well suited for research on human factors. Modern high-fidelity simulation allows very realistic interactions in very realistic environments, and video recording allows for detailed analysis of teamwork behaviors.

**Figure 1:**
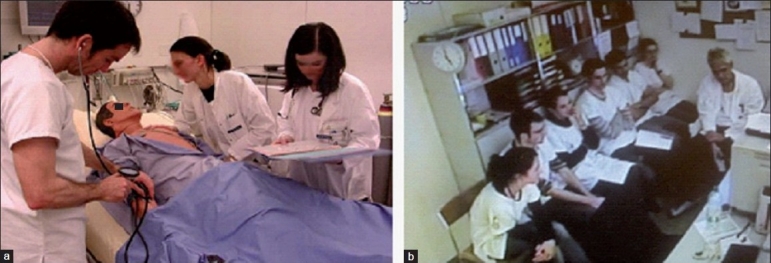
(a) Scenario of a witnessed cardiac arrest. (b) Video-assisted instruction

For example, the full body simulator used for the studies reported below is a computer-based life-sized manikin placed in a standard ICU patient room. The manikin has human physiology emulation capability and its vital signs are controlled remotely (Human Patient Simulator, METI). The manikin can talk with the care providers, inducing a personal relationship to the “patient” [Figure 1]. To increase realism, a connected monitor displays a continuous electrocardiogram and an intermittently measured noninvasive blood pressure. An intravenous catheter is placed in a peripheral vein to allow direct administration of medication. In the following, we will present some of the studies performed in this setting in the University Hospital of Basel, Switzerland, conducted by an interdisciplinary team of physicians and psychologists.

## IMPORTANCE OF FIRST RESPONDERS AND TEAM BUILDING IN RESUSCITATION

Cardiac arrest teams typically arrive at the scene with some delay. Therefore, survival of in-hospital cardiac arrests depends even more on first responders rapidly taking appropriate action than on cardiac arrest teams.[15–17] First responders face several crucial tasks: They have to diagnose the situation correctly, call for help, and start appropriate action. As help arrives on scene, the incoming professionals have to be informed about the situation, and a process of rapid team building is necessary for coordinated action.[18] Although the importance of rapid action in resuscitation is generally acknowledged, there is not much research concentrating on the initial phase of resuscitation, and the available research is mainly based on recollection of the actors. Here, simulator-based studies are of particular importance, as they allow recordings of data right from the onset of a critical situation.[18,19]

In most inpatient clinical settings, the first responder is likely to be a nurse. Previous studies suggested that nurses remained hesitant to use a defibrillator in the absence of a physician despite adequate technical training.[20] We, thus, assessed the adherence of first responders to CPR algorithms in 20 teams of nurses. Each team consisted of three nurses from the medical ICU confronted with a cardiac arrest as first responders and one resident who joined the group later.[21] We found that first responding nurses rapidly diagnosed the condition and rapidly called for help. However, in accordance with previous studies, we observed significant delays in the initiation of basic life support and defibrillation. Moreover, once commenced, chest compression and mask ventilation were executed only during approximately 60% and 75% of the total possible time. The study also showed that rapid availability of a physician (less than 1 min after the onset of the cardiac arrest), while not influencing most of the performance measures, did increase the number of countershocks administered. This study shows that measures that require coordinated activity were not initiated in a timely fashion, indicating that first responders may fail to translate their knowledge and skills into timely and effective team activity. From this study, however, it was not clear if this delay was specific for nurses as first responders.

In a second study, we therefore assessed the effects of *ad hoc* team-building on the adherence to guideline-based CPR algorithms in groups of general practitioners and hospital physicians.[22] Participants were randomized to pre-formed teams (where three physicians were present when the cardiac arrest started) or to *ad hoc* forming teams (where one physician was the first responder and the two others joined when the cardiac arrest occurred). Results showed that *ad hoc* formed teams displayed important impairments in their performance. Compared to preformed teams, *ad hoc* teams had less hands-on time, defined as uninterrupted CPR during the first 180 s of the arrest, and their first defibrillation and the administration of epinephrine were significantly delayed. Thus, independent of individuals’ skills in resuscitation, the process by which a team forms materially influences the quality of the resuscitative effort. This acquaintance process–necessary if professionals join the emergency sequentially–has to be regarded as an additional task to be resolved. These results show the important influence of human factors at the onset of an emergency, regardless of professional background.

A particularly interesting finding of this study is that *ad hoc* teams had more difficulties establishing a leadership structure than did preformed teams, as shown by the lower number of leadership utterances (for example, specific commands or task assignments) in *ad hoc* teams. More leadership utterances were, however, related to higher performance in both *ad hoc* and preformed teams. This underscores the importance of structuring the team rapidly during the early phases of a medical emergency. Studies on leadership and team coordination are, therefore, warranted.

## LEADERSHIP AND TEAM BEHAVIOR AFFECT THE QUALITY OF CARDIOPULMONARY RESUSCITATION

The importance of leadership for groups performing CPR or other emergency tasks has been suggested in several studies.[23] In an observational pioneer study, Cooper and Wakelam investigated the relationship between leadership behavior, team behavior, and task performance using video recordings of 20 real resuscitation attempts.[24] Clearer leadership of team leaders was associated not only with more efficient cooperation in the team, but also with better task performance. Interestingly, leaders who participated “hands-on” in the emergency were less likely to be efficient leaders, and team performance tended to suffer. This indicates that leadership has to be seen as a separate, effort-consuming task in resuscitation. However, this study evaluated longer lasting resuscitations and excluded short situations. The importance of leadership and communication has been recognized and evaluated in other clinical situations,[25] such as pediatric emergencies, where communication breakdowns and deficient leadership contribute to up to 70% of perinatal deaths and injuries.[26]

To evaluate the importance of leadership during the early phase of resuscitation, we assessed the relationship between leadership and performance at the onset of a simulated cardiac arrest due to ventricular fibrillation.[19] Out of 16 teams consisting of three healthcare workers each, only six were successful in that they met the performance criteria (applying two countershocks during the first 2 min, or administering two countershocks during the first 5 min provided that uninterrupted basic life support was started in less than 60 s). All teams had sufficient theoretical knowledge. Successful teams showed significantly more leadership behavior and explicit task distribution. In addition, there was a trend toward better information transfer and fewer conflicts in successful teams.

Since leadership is important and the behavior of first responders often is crucial, first-responding nurses should take leadership rapidly. Indeed, it has been shown that well-trained first-responding nurses successfully take leadership in advanced life support teams.[27] We investigated the issue of rapidly taking leadership[28] and found a positive relationship between clearer leadership behavior of the first responding nurse and resuscitation performance for the first phase of the scenario, that is, from the onset of the cardiac arrest until a physician joined the group.

In this study, however, we assumed that leadership of an event could be dynamic. Specifically, we reasoned that a change in group composition would entail the necessity to freshly establish leadership. Therefore, we investigated how leadership was transferred to an incoming resident. In all 20 groups, nurses handed over leadership to the incoming resident as soon as he or she arrived. However, not all residents took over the lead rapidly, confirming studies showing that junior doctors may lack leadership skills.[29] We also found that more leadership of the resident in the first 30 s after joining the emergency situation was related to higher performance, whereas later leadership activity was not. Thus, changes in group composition are sensitive phases that require establishing a coordinating structure. It should be mentioned that this coordination structure does not always imply that incoming professionals of higher status have to take over. Actually, our study showed that senior physicians (who entered last) supported group performance best not by taking over but by asking good questions that brought potential problems to the attention of the junior doctors.

For this reason, we recently conducted a randomized-controlled trial to assess whether teaching leadership improves team interaction and CPR performance in a high-fidelity simulated CPR.[30] After a baseline simulation and general CPR algorithm debriefing, 237 medical students were randomly assigned to receive either an additional 10 minutes leadership instruction, or a technical instruction. The leadership instruction focused on the importance of leadership and the following 4 practical items: (1) decide what to do; (2) tell your colleagues what they should do (task assignment); (3) make short and clear statements; and (4) ensure adherence to algorithm. Within this study, we found that leadership instructed students showed significantly better team performance and better overall results with regard to outcome relevant CPR measures such as beginning of CPR, hands-on time, and appropriate chest compression rate. Of note, this improvement was found after a follow-up time of 4 month documenting a sustained efficacy of this short instruction. Clearly, these findings need external validation; however, if it takes so little time to have a durable impact on CPR performance, then there should be no reason that leadership instructions should not be incorporated into standard resuscitation courses and medical school and graduate medical education curricula.[31]

## RESEARCH ON HUMAN ERRORS AND ADVERSE EVENTS USING SIMULATION

In addition to the evaluation of technical and nontechnical skills, simulator training has also been widely used for error reduction in several fields.[32–34] In recent years, the medical field has become increasingly aware of the importance of understanding the role of human factors in adverse events.[35–37] Most studies investigating human errors focus on system factors and evaluate how organizational and environmental factors lead to adverse events. Such research includes the analysis of adverse drug events,[38,39] errors in intensive care units,[40,41] and in transfusion medicine.[42] Only limited research has attempted to investigate adverse events in the context of communication and specific team interactions. There are a few studies that investigate the importance of attitudes of operating teams[43] or decision-making processes in laparoscopic procedures.[44] Some studies found that factors such as workload and communication failures were important causes of adverse events in a pediatric ICU settings.[45,46] For example, in a study simulating an emergency pediatric resuscitation, the incidence of medication errors was evaluated.[47] It was found that for 21 of the 125 medication orders (17%), the exact dose was not specified, and in 9 cases (7%) the dosage was incorrect. Only five of these dosage errors were intercepted before the drug was administered. In 9 out of 58 syringes analyzed (16%), measured drug concentrations showed a deviation of at least 20% from the ordered dose.

In our own research program, we investigated errors in two simulator studies. In the first study,[48] we examined 201 pieces of information that were transmitted in 20 groups to medical professionals that joined an ongoing simulated cardiac arrest situation. We found that 18% of the information transmitted to incoming professionals was inaccurate. Interestingly, information transmitted was more likely to be inaccurate if it was quantitative in nature and could change over time. Thus, number and strength of defibrillations and number and doses of medication were more likely to be inaccurate. However, these errors were significantly less frequent if the information was stored in memory in the same way it was needed later on (e.g., by saying “this is the third defibrillation at 300 joules”).

In the second study,[49] a diagnostic task was used. Twenty teams of three hospital physicians each were confronted with a penicillin-triggered anaphylactic shock scenario. The patient’s complaints, however, were suggestive of a tension pneumothorax. This incorrect diagnosis could be avoided, however, because the simulator manikin was programmed to display symmetrical breath sounds. We found that only 6/20 teams in the distraction version were able to correctly diagnose anaphylactic shock without help. The way participants communicated influenced the probability of correctly diagnosing the case. Groups that communicated explicitly and related different pieces of information to one another (as opposed to simply stating a diagnosis) were significantly more likely to find the correct diagnosis. The same was true for groups whose members engaged in out-loud thinking (“talking to the room”). In half of the groups, at least one physician stated hearing asymmetrical breath sounds or no breath sounds on one side, even though the breath sounds were actually symmetrical. This “auditory illusion” was present significantly more often in groups that missed the correct diagnosis and can be interpreted as an example of a confirmation bias. In none of the groups where a member wrongly observed breathing differences was he or she corrected by a colleague, even if several physicians auscultated the patient. We also established the effectiveness of our distractor by showing that, in the same scenario, 17 out of 20 groups correctly diagnosed anaphylactic shock if the distractor was not present.

These examples show that complex adverse events can be studied in simulators.

## LIMITATIONS OF SIMULATOR STUDIES

Despite the many opportunities and advantages of simulator studies in CPR research, there are important limitations. Transfer of knowledge from the simulator to the clinical setting has not been demonstrated universally.[50] Thus, studies based on simulated scenarios may be limited in generalizability. However, as outlined above, modern high-fidelity simulation has demonstrated a high degree of realism and is therefore commonly used as a central training tool in contemporary advanced life-support teaching.[4] A potential additional weakness of simulator-based studies is that participants might perceive a simulated emergency situation as less serious than a real emergency. However, in several studies, the conduct of the participants during the simulation, and while watching and discussing the video recordings of their own performance, indicated strong emotional and motivational involvement.[19,21,22,51] Another limitation of video-taped simulator sessions is that participants know that they are being monitored and thus may better adhere to CPR guidelines compared to a real-life scenario when they are not being observed (often called a Hawthorne effect). However, such an effect would bias our studies toward the null. Therefore, to the extent that guidelines are followed more closely, results such as ours are especially important in terms of detecting problems and errors. Finally, the identical conditions within simulator studies are strength and a weakness at the same time. Identical conditions improved internal reliability in intervention studies; however, the findings may just apply to the particular scenario, limiting generalizability to other situations.

## CONCLUSION

Simulator studies confirm and extend relevant findings reported in real patients. Simulator studies also provide key opportunities to study the earliest phases of resuscitation. To date, none of the key findings obtained in simulated medical emergencies contradict results obtained in studies of real emergencies. A particular strength of simulator studies is the identification of issues that are not immediately obvious for healthcare teams involved in the management of the emergency. Issues like delay in the initiation of life-saving measures or erroneous communication may have a profound impact on patients’ outcome. Our studies underline the crucial role of effective communication and good leadership behavior in managing emergency situations. If future studies validate these findings, improving team performance and leadership may translate into higher survival rates and better outcomes for patients.
